# Antimicrobial stewardship, procalcitonin testing, and rapid blood-culture identification to optimize sepsis care in critically ill adult patients: A quality improvement initiative

**DOI:** 10.1017/ash.2023.183

**Published:** 2023-06-29

**Authors:** Wendy I. Sligl, Justin Z. Chen, Xiaoming Wang, Cheyanne Boehm, Karen Fong, Katelynn Crick, Míriam Garrido Clua, Cassidy Codan, Tanis C. Dingle, Daniel Gregson, Connie Prosser, Hossein Sadrzadeh, Charles Yan, Guanmin Chen, Alena Tse-Chang, Daniel Garros, Christopher J. Doig, David Zygun, Dawn Opgenorth, John M. Conly, Sean M Bagshaw

**Affiliations:** 1 Department of Critical Care Medicine, Faculty of Medicine and Dentistry, University of Alberta and Alberta Health Services, Edmonton, Alberta, Canada; 2 Division of Infectious Diseases, Department of Medicine, Faculty of Medicine and Dentistry, University of Alberta, Edmonton, Alberta, Canada; 3 Health Services Statistical and Analytic Methods, Alberta Health Services, Edmonton, Alberta, Canada; 4 Pharmacy Services, Foothills Medical Centre, Alberta Health Services, Calgary, Alberta, Canada; 5 Pharmacy Services, University of Alberta Hospital, Alberta Health Services, Edmonton, Alberta, Canada; 6 Department of Critical Care Medicine, Cumming School of Medicine, University of Calgary and Alberta Health Services, Calgary, Alberta, Canada; 7 Division of Diagnostic and Applied Microbiology, Department of Laboratory Medicine and Pathology, University of Alberta, and Alberta Precision Laboratories, Edmonton, Alberta, Canada; 8 Department of Pathology and Laboratory Medicine, Cumming School of Medicine, University of Calgary and Alberta Health Services, Calgary, Alberta, Canada; 9 Department of Medicine, Cumming School of Medicine, University of Calgary and Alberta Health Services, Calgary, Alberta, Canada; 10 Department of Laboratory Medicine and Pathology, University of Alberta, Edmonton, Alberta, Canada; 11 Department of Pathology and Laboratory Medicine, University of Calgary, Calgary, Alberta, Canada; 12 Institute of Health Economics, Edmonton, Alberta, Canada; 13 Department of Community Health Sciences, University of Calgary, Calgary, Alberta, Canada; 14 Division of Pediatric Infectious Diseases, Department of Pediatrics, Faculty of Medicine and Dentistry, University of Alberta, Edmonton, Alberta, Canada; 15 Division of Pediatric Critical Care, Department of Pediatrics, Faculty of Medicine and Dentistry, University of Alberta, Edmonton, Alberta, Canada; 16 Critical Care Strategic Clinical Network, Alberta Health Services, Edmonton, Alberta, Canada

## Abstract

We examined the effect of an antimicrobial stewardship program (ASP), procalcitonin testing and rapid blood-culture identification on hospital mortality in a prospective quality improvement project in critically ill septic adults. Secondarily, we have reported antimicrobial guideline concordance, acceptance of ASP interventions, and antimicrobial and health-resource utilization.

Sepsis is a leading cause of hospital mortality, particularly in the intensive care unit (ICU)^1^, and the related financial costs to healthcare systems are substantial.^2^ Antimicrobial use in ICUs is considerable; early, effective antimicrobial therapy has been associated with improved sepsis outcomes. However, up to 50% of antimicrobials are suboptimally prescribed, contributing to adverse events, increased costs, and antimicrobial resistance.^3^ Antimicrobial stewardship programs (ASPs) aim to reduce these adverse outcomes through optimal prescribing practices. The use of procalcitonin testing (PCT) as an adjunctive tool is supported by guidelines.^4^

In this quality improvement study, we evaluated the impact of ASP with standardized PCT testing and rapid blood-culture identification (BCID) on clinical outcomes and health resource utilization in critically ill adult patients with sepsis.

## Methods

The study was approved by Health Research Ethics. Adult patients (aged ≥18 years) with confirmed or suspected sepsis (Sepsis-3)^5^ admitted to 2 academic, multidisciplinary ICUs in Alberta, Canada, between 2017 and 2018 were prospectively enrolled.

Each unit had a 12-week baseline period (phase 1) and an intervention period (phase 2). Clinical data were obtained through interrogation of health records and administrative databases. Clinical variables included age, sex, and comorbidities. Data regarding microbiology testing, antimicrobial therapy, and drug allergies were also collected.

Interventions included ASP, PCT, and BCID. Interventions were bundled for maximal efficiency. Only limited stewardship activities (targeting specific antimicrobials) were present in one unit prior to the study.

The ASP intervention utilized prospective audit and feedback (PAF), provided by dedicated ASP physicians and/or pharmacists. All antimicrobials prescribed were assessed 1–3 days from ICU admission and again at 3–5 days. PCT (bioMérieux, France) levels were measured daily for a maximum of 7 days or until ICU discharge. Clinical decision support, including evidence-informed stopping rules (PCT <0.25 ng/mL or 90% reduction from baseline testing) and continuation rules (>1 ng/mL or increase over time) were available to clinicians.^6^ BioFire FilmArray BCID Panel (bioMérieux, France) testing was performed on all positive blood cultures 7 days a week in parallel with standard laboratory protocols.

The primary outcome was in-hospital mortality. Secondary outcomes included clinical outcomes (Table 1), ICU antimicrobial utilization (defined daily doses [DDD] per 1,000 patient days) and health-resource utilization. Incident nosocomial *Clostridioides difficile* infections (≥72 hours and up to 90 days after ICU admission)^7^ were recorded.

**Table 1.** Secondary Outcomes

Characteristic	Preintervention (N = 342), No. (%)	Intervention (N = 385), No. (%)	OR/RR/Diff (95% CI)	*P* Value
**SOFA score**				
Mean (SD)	5.81 (3.38)	6.04 (3.72)	Diff: 0.24 (−0.29 to 0.76)	.38
Median (IQR)	5.13 (3.44–7.43)	5.33 (3.33–7.71)		
**ICDSC score**				
Mean (SD)	2.46 (1.53)	2.13 (1.48)	Diff: −0.33 (−0.55 to −0.11)	**.004**
Median	2.30 (1.10–3.50)	1.90 (1.00–3.30)		
**Delirium, no. (%)**				
Yes	329 (96.20)	367 (95.32)	OR: 0.81 (0.39–1.67)	.56
No	13 (3.80)	18 (4.68)		
**Mechanical ventilation, no. (%)**				
Yes	259 (75.73)	259 (67.27)	OR: 0.66 (0.48–0.91)	**.01**
No	83 (24.27)	126 (32.73)		
**Duration of ventilation, days**				
Mean (SD)	6.45 (6.84)	6.52 (9.85)	RR: 1.01 (0.85–1.20)	.90
Median	3.67 (1.82–8.72)	3.58 (1.48–7.52)		
**Ventilator-free days**				
Mean (SD)	4.41 (5.17)	4.76 (8.54)	RR: 1.08 (0.93–1.25)	.31
Median	2.93 (1.37–5.70)	3.01 (1.48–5.15)		
**Vasoactive support, no. (%)**				
Yes	96 (28.07)	133 (34.55)	OR: 1.35 (0.99–1.86)	.06
No	246 (71.93)	252 (65.45)		
**Duration of vasoactive support, days**				
Mean (SD)	2.21 (1.99)	2.21 (2.15)	RR: 1.00 (0.80–1.26)	.99
Median	1.53 (0.78–3.26)	1.61 (1.01–2.51)		
**RRT, no. (%)**				
Yes	49 (14.33)	47 (12.21)	OR: 0.83 (0.54–1.28)	.40
No	293 (85.67)	338 (87.79)		
**Duration of RRT (d)**				
Mean (SD)	4.33 (3.02)	4.21 (3.58)	RR: 0.97 (0.72–1.30)	.85
Median	3.42 (1.97–6.15)	3.00 (1.97–4.97)		
**ICU length of stay (d)**				
Mean (SD)	9.17 (9.12)	8.70 (12.89)	RR: 0.95 (0.83–1.08)	.43
Median (IQR)	5.96 (3.29–11.31)	5.13 (2.83–9.08)		
**Hospital length of stay (d)**				
Mean (SD)	32.59 (47.28)	27.53 (31.36)	RR: 0.84 (0.74–0.97)	**.02**
Median (IQR)	20.13 (10.11–40.10)	16.62 (8.80–33.47)		

Note. OR, odds ratio; RR, relative risk ratio; IQR, interquartile range; SD, standard deviation; SOFA, sequential organ failure assessment; ICDSC, intensive care delirium screening checklist; RRT, renal replacement therapy; ICU, intensive care unit.

To examine the relationship between our intervention and outcomes, we built logistic regression models for binary responses and negative binomial regression models for duration and frequency responses. Models were adjusted for preselected, clinically relevant variables; time and month; and interaction between our intervention and time. All statistical analyses were performed using Statistical Analysis System (SAS) Enterprise Guide version 7.1 software (SAS Institute, Cary, NC).

## Results

In total, 727 patients were included: 342 in phase 1 and 385 in phase 2. The mean age was 58 (±15) years, and 58% were male, The mean APACHE II score was 24 (±8), and the mean sequential organ failure assessment (SOFA) score was 9 (±4). Also, 71% were mechanically ventilated and 32% received vasoactive support.

There was no significant difference in hospital mortality between phases (25.4% vs 26.5%; adjusted odds ratio [aOR], 1.06; 95% CI, 0.76–1.47; *P* = .75). There were no significant differences in clinical outcomes (Table 1). Also, there were no significant differences in utilization of mechanical ventilation, vasoactive support, or renal-replacement therapy. Length of ICU stay was similar; however, hospital stay was shorter in phase 2 (mean difference, 5.06; 95% CI, 4.46–5.84; *P* = .017).

Antibacterial utilization was significantly lower in phase 2; with an overall 7.3% reduction (*P* = .010). The largest reduction in utilization of any 1 antimicrobial was 17.6% (piperacillin-tazobactam, *P* = .038). There were no significant differences in incident nosocomial *C. difficile* infections: 3.5% of patients or 10.9 cases per 10,000 patient days in phase 1 versus 2.1% or 5.7 cases per 10,000 patient-days in phase 2 (*P* = .24).

The ASP team conducted 651 patient assessments and reviewed 1,279 antimicrobial prescriptions. Of 998 empiric prescriptions, 86% were guideline concordant. Overall, 64% of prescriptions were adjudicated as appropriate (Table 2). In prescriptions assessed as inappropriate, ASP recommended discontinuing the agent in 29% of cases and changing the prescription in 51%. ASP recommendations were fully accepted by ICU clinicians in 93% of assessments. In total, 1,839 PCT measurements were performed, with levels ranging between 0.05 and 1046 ng/mL. Also, 14% of initial PCT values and 15% of all values met the <0.25 ng/mL stopping rule. Of the 81 patients with a PCT value <0.25 ng/mL, 36% had antimicrobials discontinued.

**Table 2.** Antimicrobial Stewardship Recommendations

Variable	Total Prescriptions (N = 1259)	Prescriptions at Initial PAF (N = 770)	Prescriptions at Follow-Up PAF (N = 489)
Appropriate, no change, no. (%)	808 (64.2)	531 (65.7)	277 (34.3)
**Discontinue agent, no. (%)**	132 (10.5)	65 (49.2)	67 (50.8)
Redundant therapy	12	6	6
Not an infection	19	3	16
Not indicated	101	56	45
**Change agent, no. (%)**	116 (9.2)	65 (56.0)	51 (44.0)
Less broad-spectrum agent(s)	70	36	34
More broad-spectrum agent(s)	25	17	8
Less expensive agent(s)	3	2	1
Less toxic alternative agent(s)	5	1	4
More effective agent(s)	13	9	4
**Change regimen, no. (%)**	114 (9.1)	76 (66.7)	38 (33.3)
Improper dosage for indication	32	29	3
Improper dosage for renal function	25	16	9
Improper dosage for weight	10	9	1
Improper route	20	17	3
Improper dosing interval	3	1	2
Improper duration	24	4	20
Other, no. (%)	89 (7.1)	33 (37.1)	56 (62.9)

Note. PAF, prospective audit and feedback.

Overall, 44 bloodstream infections were identified (6%), including 40 monomicrobial infections, 17 species and 49 unique isolates. BCID identified 13 of 17 species. The most common gram-positive organism was *Staphylococcus aureus* (7 MSSA and 3 MRSA isolates) and most common gram-negative organism was *Escherichia coli* (7 isolates). BCID species identification was more rapid (mean, 20.4 ± 15.9 hours) than standard laboratory testing in 71% instances. BCID preliminary antimicrobial susceptibility testing was 28.6 ± 12.1 hours faster in 10 of 49 isolates; all of which were *S. aureus* or *Enterococcus faecium*.

## Discussion

This prospective, real-world, pragmatic pre- and postimplementation quality improvement project in critically ill patients with sepsis revealed no difference in mortality but reduced overall antimicrobial utilization. Our findings support that ASP interventions do not negatively impact survival in critically ill patients with sepsis and can significantly reduce antibacterial utilization. This observation is supported by other studies.^8–10^

Although we did not detect a significant difference in mortality, hospital length of stay was shorter with our intervention. This may be the result of more effective antimicrobial therapies, shorter individual durations of therapy and discontinuation of antibiotics when not necessary.

We found high clinical-practice guideline concordance with empiric antimicrobial prescribing. ASP assessments revealed opportunities for antimicrobial optimization in 35% of patients. Acceptance of recommendations was high, and a significant reduction in overall antibacterial utilization was observed.

PCT aided in antimicrobial streamlining and/or discontinuation and may have increased confidence in decision making for clinicians. Moreover, 15% of all PCT values were <0.25 ng/mL, leading to antimicrobial discontinuation in 36% of instances.

Few patients (6%) were bacteremic. BCID identified species in 76% and did so more rapidly in 71% of instances compared to standard laboratory protocols. Preliminary susceptibilities were available 28.6 hours sooner compared to standard laboratory testing, mostly for drug-resistant organisms such as MRSA and VRE.

The strengths of this study include its large sample size, prospective data collection, and completeness of follow-up. In addition, we used an objective clinical definition of sepsis.

Our study had several limitations. We enrolled patients from 2 large, adult, academic ICUs without comprehensive ASPs, which may not be representative of smaller and/or community ICUs or units with established ASPs. We performed a pre- and postintervention analysis; hence, no causal inference can be made, and the results may be subject to unmeasured bias. There were also outcomes with few events, such as *C. difficile* infections and episodes of bacteremia. Additionally, our results may not be generalizable to other healthcare systems, and the individual effects of each intervention were not assessed.

In conclusion, the implementation of bundled ASP, PCT, and BCID did not affect mortality in critically ill patients with sepsis but was found acceptable and safe and aided in reducing and rationalizing antimicrobial therapy. Based on these findings, we advocate for ASPs in all ICUs providing care for septic patients. Adjunctive PCT measurements may be useful in further risk-stratifying patients and increasing clinician confidence for antibiotic discontinuation. Rapid blood-culture technologies, specifically BCID, may provide rapid species identification and preliminary antimicrobial susceptibility results, but BCID requires further study in a larger population.
